# Influence of CNT Incorporation on the Carbonation of Conductive Cement Mortar

**DOI:** 10.3390/ma14216721

**Published:** 2021-11-08

**Authors:** Gun-Cheol Lee, Youngmin Kim, Soo-Yeon Seo, Hyun-Do Yun, Seongwon Hong

**Affiliations:** 1 Department of Architectural Engineering, Korea National University of Transportation, Chungbuk, Chungju 27469, Korea; gclee@ut.ac.kr (G.-C.L.); imkym97@ut.ac.kr (Y.K.); syseo@ut.ac.kr (S.-Y.S.); 2 Department of Architectural Engineering, Chungnam National University, Daejeon 34134, Korea; wiseroad@cnu.ac.kr; 3 Department of Safety Engineering, Korea National University of Transportation, Chungbuk, Chungju 27469, Korea

**Keywords:** multi-walled and single-walled carbon nanotubes, carbonation, electrical resistance, porosity, strength

## Abstract

This study analyzed the influence of carbon nanotubes (CNTs) on the carbonation conductive cementitious composites. Two powder types of CNT, multi-walled and single-wall CNTs, were employed to give the cement mortar the conductivity, and four tests including the accelerated carbonation, compressive and flexural strength, electrical resistance, and porosity tests were carried out. To intentionally accelerate the carbonation, the prismatic specimens of conductive cement composites were fabricated and stored in the controlled environmental chamber at a constant temperature of 20 ± 2 °C, constant relative humidity of 60 ± 5%, and carbon dioxide (CO_2_) concentration of 5% for 12 weeks. It was observed that carbonation resulted in only chemical damage so that there was no change in the electrical resistance value of conductive cementitious mortar that had undergone a carbonation attack.

## 1. Introduction

The quality degradation of concrete structures is generally caused by the continuous occurrence of various types of environmental factors [1]. In particular, the carbonation problems of concrete structures that are common phenomena in metropolitan areas, seaside cities, or underground spaces have been gradually raised since the 1980s, and the durability due to the carbonation has significantly decreased as the result of global climate changes [2,3,4,5,6,7,8,9]. In general, carbonation is the result of a chemical reaction in which calcium hydroxide, a hydrate in the concrete structures, is changed into calcium carbonate as carbon dioxide (CO_2_) diffuses, and carbonation lowers the pH in concrete or cementitious composites, leading to neutralization. As the carbonized concrete or cementitious composite is neutralized, the reinforcing bars inserted inside reinforced concrete structures are prone to corrosion, and the corroded reinforcement generates internal cracks due to its volume expansion. Furthermore, as this process progresses for long periods of time, such cracks that occur on the surface of the structure pose a great threat to structural safety. Particularly, underground structures, such as a subway structure, are exposed to a CO_2_ concentration of more than 660 ppm and high humidity at 60–70%, which promotes carbonation [10,11].

Recent research in the fields of concrete structure has been focused on the development of structural health monitoring systems (SHM) using self-monitoring or self-sensing composites [12]. Choi E.K. et al. [13] used steel fibers both to embed the self-sensing system into the concrete structures and to strengthen the tensile strength. In addition to the incorporation of steel fiber in concrete mixtures, using carbon nanotubes (CNTs) has been investigated by many researchers and scholars because CNTs can give the concrete composites the conductivity [14,15,16]. Generally, the dosage of CNTs was up to 2.0% of the weight of the binder because of its low density of 1.3–1.4 g/cm^3^ [17,18]. Moreover, poor dispersion of CNTs in the composites induced by van der Waals forces between the CNT particles has become a major issue because it caused low mechanical performance of the concrete structures [19]. To address this problem, many studies have been conducted. Collins F. et al. [20] used aqueous solutions containing CNTs with several types of admixtures such as air-entraining agents based on alkylbenzene sulfonic acid, styrene butadiene rubber copolymer latex, and aliphatic propylene glycol ether including ethoxylated alkylphenol, polycarboxylate, calcium naphthalene sulfonate, naphthalene sulphonic acid derivative, and lignosulfonate. Sobolkina A. et al. [21] investigated the effects of sonication on CNT dispersion with anionic and nonionic surfactants. Various surfactants including cetrimonium bromide, sodium dodecyl benzene sulfonate, and triton X-100 were used as admixtures to uniformly disperse the CNTs in the mixtures [22]. To develop the appropriate dispersion of CNT in cementitious composites, Gao et al. [23] added graphene oxide and employed an ultrasonication technique. As the CNT mixtures have been uniformly dispersed, it has not only proper strength but also excellent self-sensing performance. Despite numerous published articles, investigations, and studies on the carbonation of concrete structures and SHM using CNT above, to the authors’ knowledge, at present there is no strong consensus in the literature regarding the analysis of carbonated conductive cementitious composites. To bridge this gap, physical, mechanical, and electrical characteristics of CNT incorporated cementitious mixtures that have undergone carbonation attack are first obtained through various experiments, and the data and results of experiments can be used to update how carbonation influences the performance of conductive cementitious composites.

The remainder of this research is organized as follows; the ordinary Portland cement (OPC), standard sand, and two powder types of CNT used in the experiment were first analyzed in terms of physical and chemical properties, the mixture proportions of conductive cementitious composites were explained in detail, four experiments including compressive and flexural strength, electrical resistance, and porosity tests were conducted to investigate the CNT effects on the carbonation and finally, through the data from electrical resistance and porosity experiments, meaningful findings were reached.

## 2. Experimental Program

### 2.1. Materials

**Figure 1:**
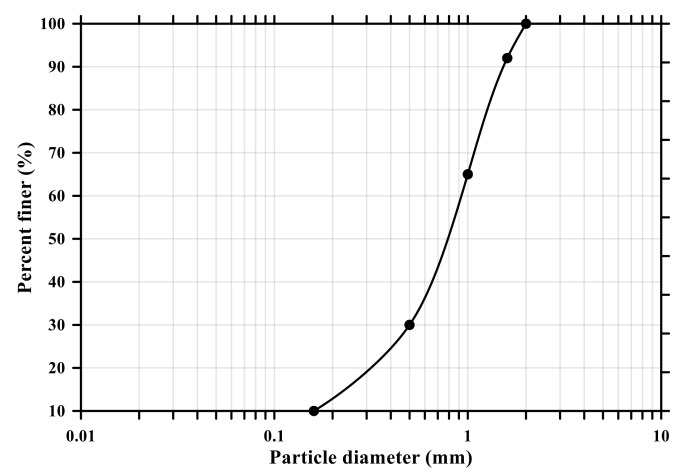
Particle size distribution curve of standard sand (KS L ISO 679).

**Figure 2:**
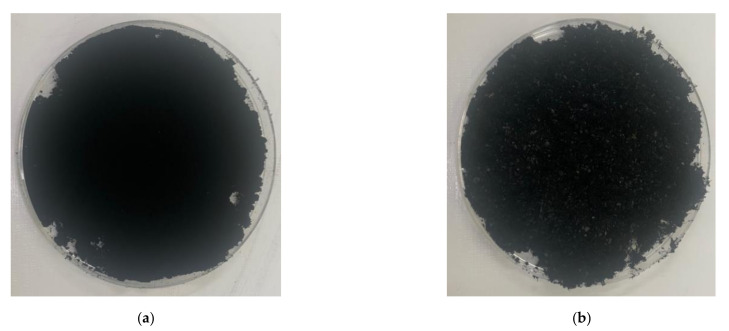
Picture of powder-type carbon nanotubes: (**a**) multi-walled carbon nanotubes; (**b**) single-walled carbon nanotubes.

In this experiment, ordinary Portland cement (OPC, Type I KSL 5201 [24]) and standard sand (KS L ISO 679 [25]) were employed. Table 1 and Figure 1 show the chemical and physical properties of OPC and particle size distribution curve of standard sand (KSL ISO 679 [25]), respectively. To make conductive composites, two powder types of CNTs, multi-wall CNT (MW) and single-wall CNT (SW) (Tuball, OCSiAI, Leudelange, Luxembourg), were added to the mixtures and presented in Figure 2, and their physical porosities are summarized in Table 2.

### 2.2. Mixture Proportions

The mixture proportions are shown in Table 3. The CNTs used in this study are divided into MW and SW and prepared at three levels (0, 1.0, and 2.0% mass fraction), which are determined based on the literature review [17,18]. The specimens were fabricated in accordance with KS L ISO 679 [25]. Admixture (poly carboxylate-based high-performance water reducing agent, KS F 2560 [26]) was added to the mixture to improve workability and homogenous dispersion, and its amounts at MW 1.0, SW 1.0, MW 2.0, and SW 2.0 were 2.0, 6.0, 4.0, and 14.0%, respectively.

### 2.3. Experimental Method

**Figure 3:**
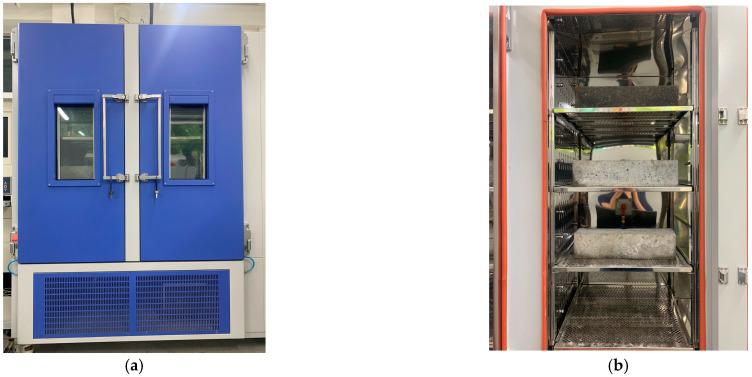

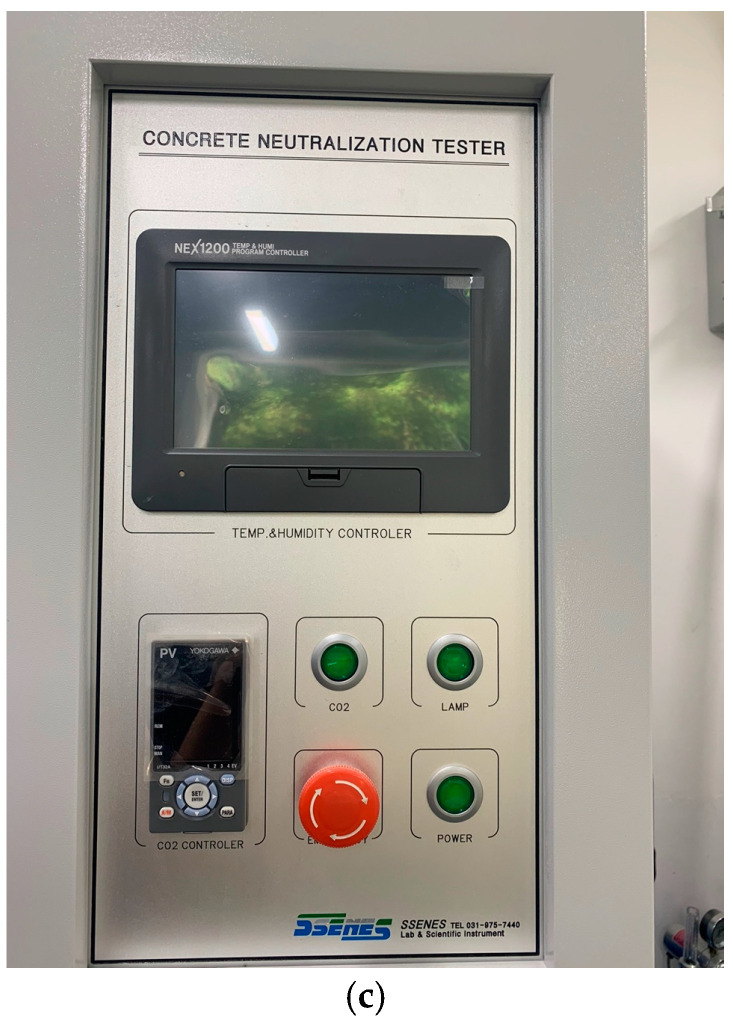
Carbonation acceleration chamber: (**a**) outside chamber; (**b**) inside chamber; (**c**) control system.

To analyze the conductive properties of the cement mortar incorporating CNTs undergone carbonation, the accelerated carbonation, compressive and flexural strength, electrical resistance, and porosity tests were carried out. External factors affecting the carbonation of cementitious composites are generally temperature, humidity, and carbon dioxide concentrations. In this study, to accelerate the carbonation of the conductive cement mortar, the prismatic specimens with a cross-section of 100 × 100 mm^2^ and a length of 400 mm were fabricated and stored in a carbonation acceleration chamber (SSENES Lab & Scientific Instrument, NEX1200) at a constant temperature of 20 ± 2 °C, constant relative humidity of 60 ± 5%, and CO_2_ concentration of 5% until the target age (up to 12 weeks), as demonstrated in Figure 3. The control specimens with the same dimension were cured in a water tank at 20 ± 2 °C for the same number of days. The specimens were cut into two halves and sprayed with a 1% phenolphthalein solution on the measurement surface, and the carbonation depth was then determined by measuring the distance from the edge of the specimen to the color boundary in accordance with the carbonation depth measurement method of cementitious composites (KS F 2596 [27]). The carbonation velocity coefficient (A) was calculated based on Equation (1).

(1) $$x_{c}^{}=A\sqrt{t}$$

where x_c_ is carbonation depth (mm), and t is accelerated carbonation period (week).

**Figure 4:**
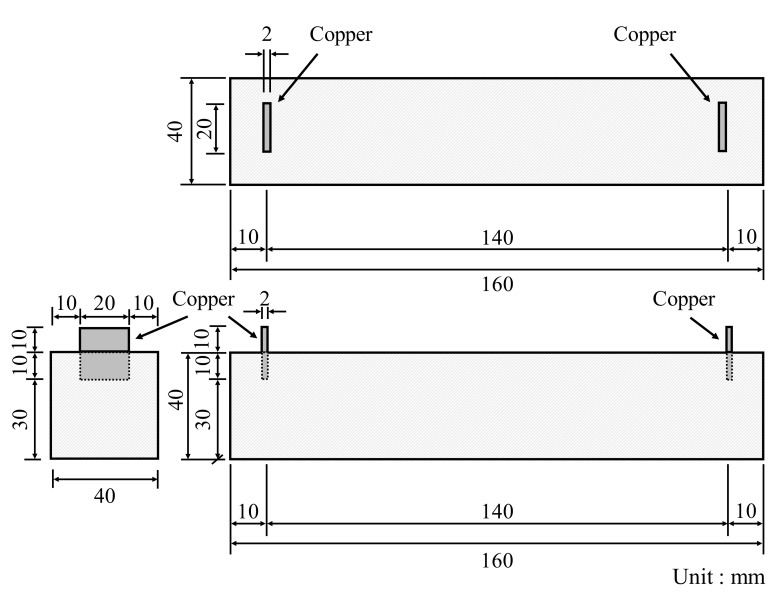
Specimen dimension of conductive cement mortar for electrical resistance measurement.

In accordance with KS L ISO 679 [25], three prismatic specimens with a cross-section of 40 × 40 mm^2^ and a length of 160 mm were fabricated, demolded after 24 h, and cured in a water tank maintained at 20 ± 1 °C until the target day. The compressive and flexural strengths were measured at 3, 7, and 28 days. Figure 4 displays the prismatic specimen dimensions of conductive cement mortar for the electrical resistance test, which were the same dimension as the strength tests. To properly measure the resistance, copper plates were installed at both ends of the specimen and a two-probe method with the DAQ970A data acquisition system with the BenchVue program was used and alternating current (AC) was used. Due to the moisture content effect of cementitious composites [28], the specimens were dried in an environmental chamber for 24 h at a temperature of 80 ± 1 °C. After drying, 5 V AC power was supplied to the copper plates at both ends of the cementitious composites. To stabilize the resistance value, it was measured approximately 20 min after the supply of current. Since pore size and its distribution can influence mechanical properties of conductive cementitious composites which have undergone a carbonation attack, pore distribution curves are necessarily obtained [29,30,31]. For this, approximately 2 g of sample was collected from the top surface of the specimen, and mercury porosimetry analysis was performed by using the Mercury Porosimeter (ATS Scientific Inc., Autopore V 9600, Burlington, ON, Canada). The pressure range of Autopore V 9600 is between 50 and 60,000 psi and it can measure pore sizes of 0.003–900 μm.

## 3. Test Results and Discussion

### 3.1. Carbonation Depth of Conductive Cement Mortar

**Figure 5:**
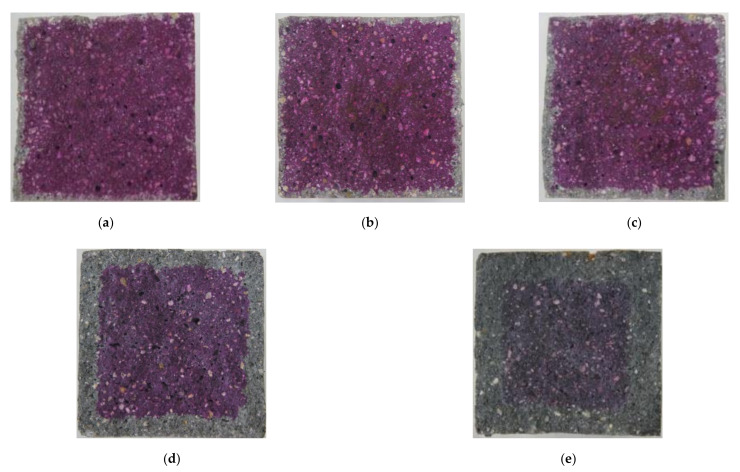
The results of carbonation of conductive cement mortar (12 weeks): (**a**) plain; (**b**) MW 1.0; (**c**) MW 2.0; (**d**) SW 1.0; (**e**) SW 2.0.

Carbonation occurs in the cement mortar as the calcium hydroxide (Ca(OH)_2_) in cement reacts with CO_2_ from the atmosphere and water in the pore. First, carbon acid (H_2_CO_3_) is the result of a reaction between carbon dioxide and water. The generated carbon acid reacts with the calcium hydroxide to form calcium carbonate (CaCO_3_) and water, as shown in Equation (2). In general, the volume of calcium carbonation increases by about 11.7% compared to that of calcium hydroxide. Consequently, as carbonation proceeds in plain cement mortar, the carbonated region forms a dense pore structure resulting in the prevention of the CO_2_ penetration and restraint of the CO_2_ effect [32,33]. However, the conductive cement mortar has a larger pore than that of plain composites so that more diffusion of CO_2_ in the CNT cementitious composites through these pores takes place. Figure 5 shows the results of carbonation of conductive cement mortar assessed by using alkalinity indicator, Phenolphthalein, after 12 weeks of the accelerated carbonation test. It was confirmed that CNT cementitious materials accelerated the carbonation.

Ca(OH)_2_ + H_2_CO_3_ → CaCO_3_ + 2H_2_O (2)

**Figure 6:**
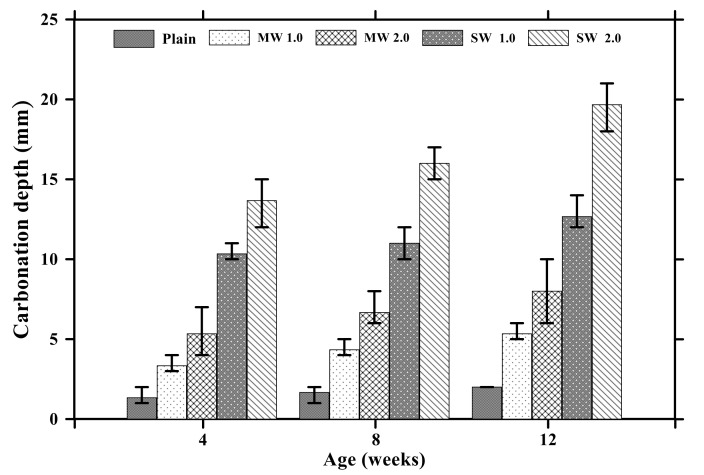
Carbonation depths of conductive cementitious composites after carbonation.

Figure 6 provides the results of carbonation depths of conductive cement mortar after the carbonation attack. In the case of the plain specimen, the carbonization depth increased to 2 mm with no coefficient of variation (COV) for 12 weeks. The depth of the MW 1.0 and MW 2.0 specimens was 5.3 mm with COV of 11% and 8 mm with COV of 25%, respectively, for 12 weeks whereas the carbonation depth of SW 1.0 and SW 2.0 was observed to be 12.6 mm with COV of 9% and 19.6 mm with COV of 8%, respectively, for 12 weeks. It signified that CNT clearly influenced accelerating the carbonation of conductive cement mortar.

**Figure 7:**
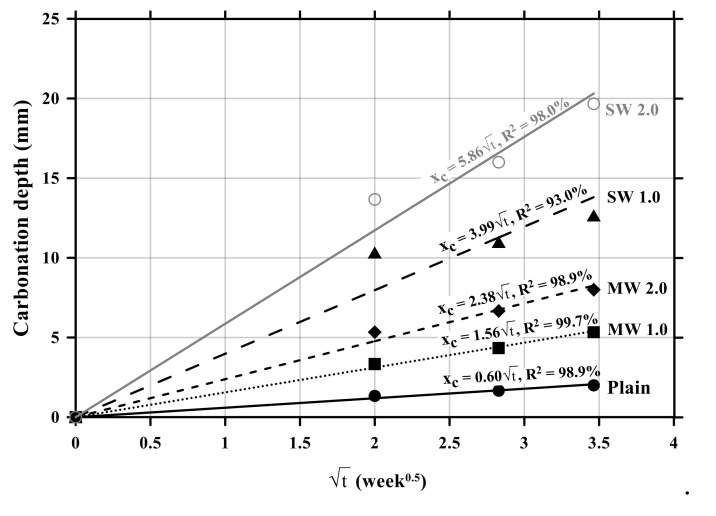
Relationship between carbonation depths and time of conductive cement mortar.

Figure 7 exhibits the relationship between carbonation depths and time. The carbonation velocity coefficient was 0.60 with an R-squared (R^2^) of 98.9% for the plain specimen without CNTs. The carbonation velocity coefficient of the MW 1.0 and MW 2.0 specimens was measured to be 1.56 with an R^2^ of 99.7% and 2.38 with an R^2^ of 98.9%, respectively, while that of SW 1.0 and SW 2.0 was 3.99 with an R^2^ of 93.0% and 5.86 with an R^2^ of 98.0%, respectively. It was observed that the carbonation velocity coefficient of SW was 250% greater than that of MW and the carbonation rate of conductive cement mortar increased by 150% as the amount of CNT was doubled in the mixture. Therefore, it was confirmed that the incorporation of CNTs accelerated the carbonation of cement mortar.

### 3.2. Compressive and Flexural Strength of CNT Cementitious Materials

**Figure 8:**
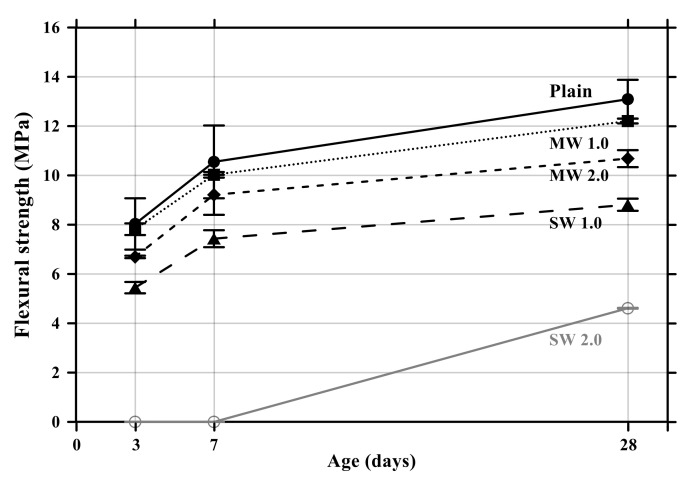
Flexural strength of conductive cementitious composites.

**Figure 9:**
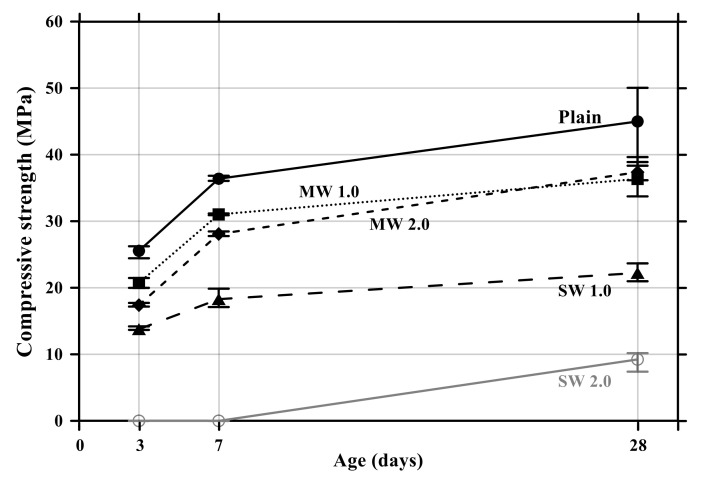
Compressive strength of conductive cement mortar.

The flexural and compressive strength results of conductive cement mortar are shown in Figure 8 and Figure 9, respectively. The specimens incorporating CNTs showed a further decrease in compressive and flexural strengths compared with the plain specimen. Particularly, when the amount of incorporation of CNTs increased, the compressive and flexural strengths decreased because CNT is a hydrophobic composite, so it is impossible to be dispersed properly in the mortar mixtures, and it exists as pores in the composites, which results in decreases in the mechanical performance of CNT-embedded cementitious composites. This is also the reason why conductive cement mortar accelerated carbonation. Since SW 2.0 was not completely cured by the age of 7 days, its strength was zero. Because excessive chemical admixture (14%) was used, it was considered that it delayed the curing. It was clearly observed that compressive and flexural strength decreased as the amount of CNT incorporation increased, and the decrease of SW was greater than that of MW.

### 3.3. Electrical Resistance Properties of Conductive Cementitious Composites

**Figure 10:**
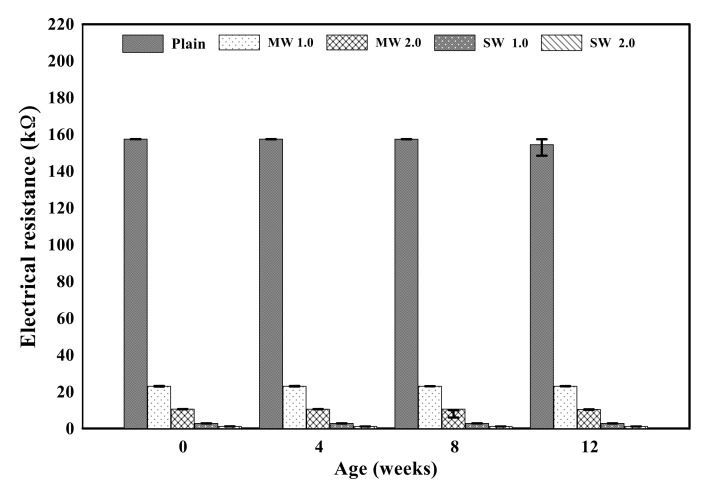
Electrical resistance of conductive cement mortar before and after carbonation.

Figure 10 displays the electrical resistance properties of conductive cementitious before and after the accelerated carbonation. The electrical properties of the specimen before deterioration damage showed that as the CNTs were incorporated, the resistance value suddenly decreased to about 80–90%. By comparing MW with SW, it was clearly measured that the electrical resistance of SW was lower than that of MW. Since CNTs are conductive nanomaterials, conductivity can be given to the cement mortar and the small resistance is measured in spite of the bundle phenomenon induced by the van der Waals force in the composites. In other words, cement mortar mixing with CNTs has the capability to exhibit electrical performance. In addition, it was found that there was no significant change in the electrical resistance value as carbonation proceeded. The carbonation of cementitious composites did not result in internal damage, but it was a chemical change in which calcium hydroxide reacts with CO_2_ to produce calcium carbonate, which made only dense pore structures in the mixtures. It signified that the carbonation of conductive cementitious materials caused only mechanical damage so that there was no change in electrical performance.

### 3.4. Pore Distribution Characteristics of Conductive Mortar

**Figure 11:**
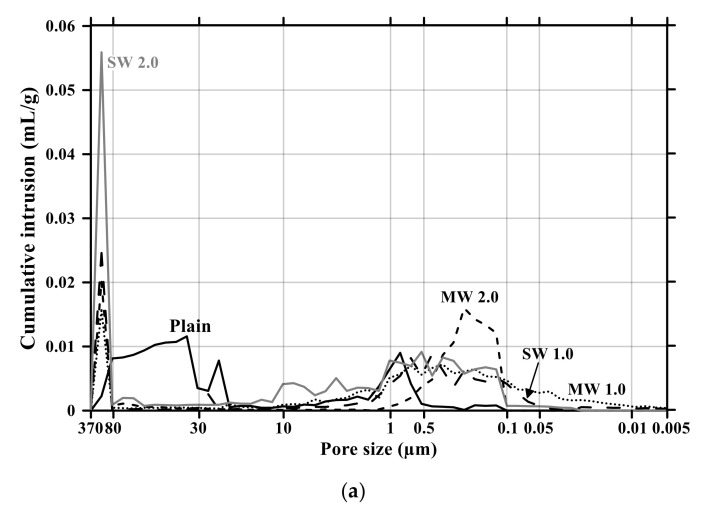

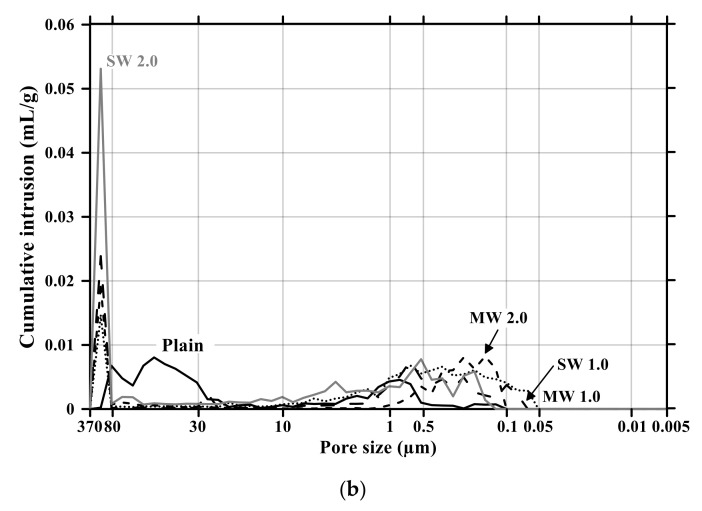
Pore size distribution of conductive cementitious composites before and after carbonation: (**a**) before carbonation and (**b**) after carbonation.

**Figure 12:**
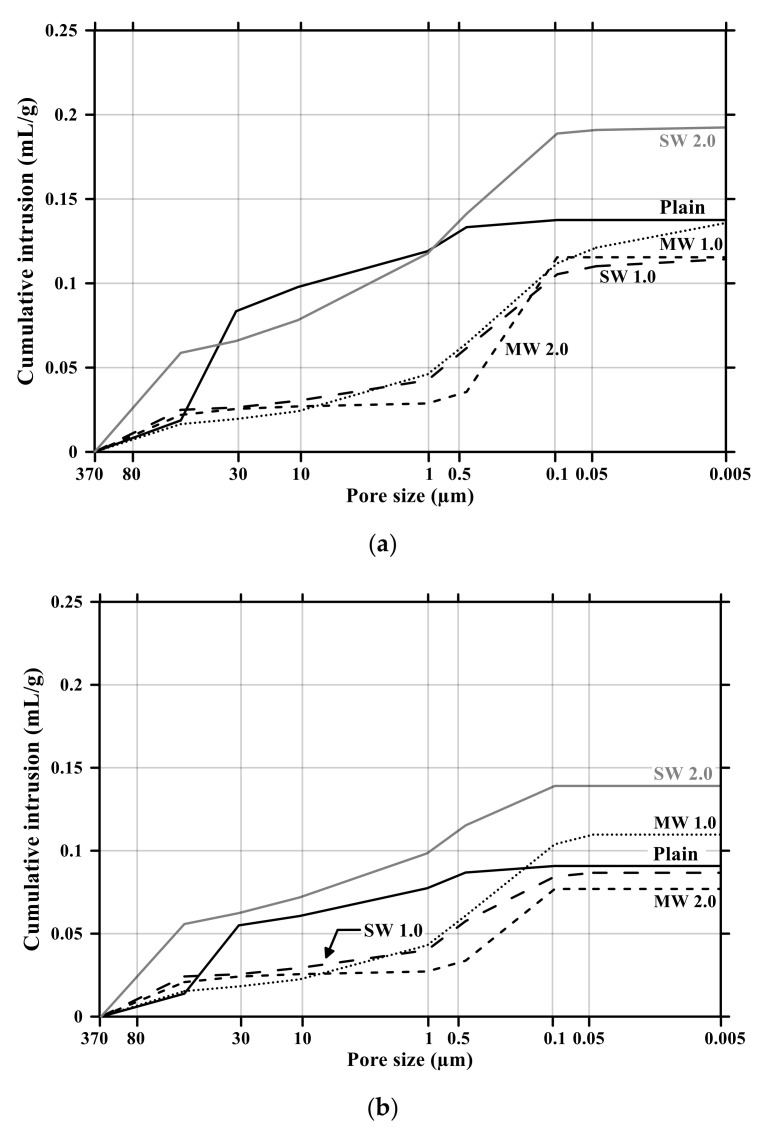
Cumulative pore volume of conductive cement mortar before and after carbonation: (**a**) before carbonation and (**b**) after carbonation.

Figure 11 and Figure 12 present the pore size distribution and its cumulative pore volume of the conductive cementitious specimen before and after the carbonation attack, respectively. Before the carbonation in Figure 11a, it was observed that relatively large pores with sizes ranging between 370 μm and 80 μm increased with increasing the dosage of CNTs. In particular, in the range from 370 μm to 80 μm, the largest cumulative pore volume of SW 2.0 specimens was measured (see Figure 11a and Figure 12b) and the pore size of conductive cement mortar incorporating SW was relatively larger than that of the composites with MW, which are clearly different pore size characteristics from the plain specimen. The filling effect due to the diameter of 5–100 ηm and 1.2–3.0 ηm for MW and SW, respectively, resulted in no pore distribution between 0.1 μm and 0.05 μm for MW 2.0 and SW 2.0. However, it was obviously observed that the specimen of MW 1.0 and SW 1.0 had pores from 0.1 μm and 0.05 μm. These are because that CNTs were hydrophobic and difficult to be uniformly distributed in the composites. In the case of plain mortar, the pore size was between 370 μm and 35 μm and there were no micro-pore characteristics. These pores from 370 μm to 35 μm were considered to be the ones left as the remaining water evaporated after the water reacted with cement. Figure 11b provides the pore distribution curve of the cementitious composites after carbonation. The maximum pore distribution of the conductive mortar was the same as ones before the carbonation attack. Carbonation of concrete structure implied loss of alkali and it became neutralization. Carbonation did not result in the internal and/or external damage of the concrete structures caused by freeze–thaw or sulfuric acid erosion. Instead of physical change, the generated calcium carbonate caused the filling effect as shown in Figure 11 and Figure 12. Therefore, it was considered that no physical damage occurred in the conductive cementitious composites that had undergone carbonation so that the connection between CNTs was maintained and conductivity was not physically damaged. Moreover, as the CNT incorporation dosage increased, the distribution of large pores was measured. This is the evidence supporting the above results of compressive and flexural strength as well.

## 4. Conclusions

To evaluate the change in the conductivity of the cementitious mortar incorporating MW and SW due to the carbonation, four laboratory tests such as the accelerated carbonation, compressive and flexural strength, electrical resistance, and porosity tests were performed and the following findings were drawn;

1. The acceleration rate of carbonation of conductive cementitious composite increased with an increasing amount of incorporation of CNTs because the large pores generated from the incorporation of CNTs facilitated the penetration of CO_2_ in the mortar. It was found that the carbonation velocity coefficient of SW was 2.5 times greater than that of MW and the carbonation rate of conductive cement mortar increased by 1.5 times as the dosage of CNT was doubled in the mixture.
2. When CNTs were mixed with the cement mortar, the compressive and flexural strengths decreased compared to those of the plain mortar due to an increase in the internal pore volume. In particular, it was measured that relatively large pores with sizes ranging from 370 μm to 80 μm occurred due to the van der Waals force resulting from the incorporation of CNTs. These pores resulted in the degradation of mechanical properties.
3. The electrical resistance value of the conductive cement mortar was about 10–20% of the plain specimen, signifying that it had conductivity performance. In addition, the decrease in resistance value was greater in SW than in MW, indicating that SW had better electrical properties than NW. Furthermore, there was no significant change in the electrical properties due to the carbonation. It denoted that carbonation only led to chemical change without causing any physical damage to the inside of the cement mortar, and the connection of CNTs was thus unimpaired.
4. Through the test results of the pore distribution curve, it was worth noting that large pores with sizes ranging between 370 μm and 80 μm increased with the increase in the amount of CNTs. The filling effect due to the diameter of 1.2–100 ηm for CNTs caused no micro-pore distribution in a range between 0.1 μm and 0.05 μm for MW 2.0 and SW 2.0, while it was obviously detected that the MW 1.0 and SW 1.0 composites had micro-pores. It implies that hydrophobic CNTs were difficult to be uniformly dispersed in the mixtures and CNT incorporation clearly resulted in a decrease in the mechanical performance of cement mortar. After carbonation the pore distribution curves were clearly changed because the pore created by CNTs would be the penetration route of CO_2_ into the inside of cementitious composites, causing acceleration of carbonation. The generated calcium carbonate resulted in the filling effect and chemical change in the composites such that the connection between CNTs were not damaged and the conductive cementitious composite had a self-sensing performance.

## Figures and Tables

**Table 1:** Properties of ordinary Portland cement.

Chemical Properties (%)					Physical Properties	
SiO_2_	Al_2_O_3_	Fe_2_O_3_	CaO	MgO	Density (g/cm^3^)	Specific Surface Area (cm^2^/g)
22.23	5.21	3.38	64.58	2.3	3.15	3300

**Table 2:** Physical properties of multi-walled and single-walled carbon nanotubes.

	MW	SW
Electrical resistance (Ω·m^2^)	5.1 × 10^−6^	10 × 10^−4^
Diameter (nm)	5–100	1.2–3.0
Length (μm)	10	10
Specific surface area (m^2^/g)	130~160	700~900
Tension (GPa)	<50	45
Thermal conductivity (W/m·K)	3000	6000

**Table 3:** Mixture proportion of conductive cement mortar.

Sample	W/C (%)	Weight (g)			
		Cement	Water	Sand	CNT
Plain	50	450	255	1 350	0
MW 1.0 or SW 1.0					4.50
MW 2.0 or SW 2.0					9.00

## Data Availability

Data contained within the article.
